# Factors of variability in the accumulation of waste in a mountain region of southern Poland

**DOI:** 10.1007/s10661-020-8103-y

**Published:** 2020-01-31

**Authors:** Grzegorz Przydatek, Klaudia Ciągło

**Affiliations:** Engineering Institute, State University of Applied Sciences in Nowy Sącz, Zamenhofa 1a St, 33-300 Nowy Sącz, Poland

**Keywords:** Rate accumulation, Collection, Community, Waste management

## Abstract

The analysis of change factors in waste management in the period from 2012 to 2015 in three municipalities in a mountainous region of southern Poland exhibited strong differentiation. This was recognised based on multiple indicators of waste accumulation. Such differences were investigated between two periods, which highlighted the effect of changes in waste management primarily resulting from Directive 2008/98/EC. These changes relate to the development of selective waste collection based on eight types of waste. On this basis, an increase was observed in the amount of waste collected in the communities. Particular attention was paid to the community with the highest number of tourists, the waste accumulation rate (452.74 kg per capita) and the cost per year. An increase in the number of tourists is an important factor in terms of waste accumulation, and other factors made it possible to recognise the direction of the changes taking place in waste management. In terms of changes in waste accumulation, the implementation of the European Union law into the national law in the field of waste management is a significant factor.

## Introduction

Waste can be categorised as hazardous or non-hazardous and inert. Non-hazardous waste includes kitchen waste, garden waste, paper and cardboard, textiles, metals and similar materials used in everyday life (Kawai and Tasaki 2016). Growth in terms of population, economy and the standard of living has contributed to by-products in the form of an increasing amount of municipal solid waste (MSW) (Akdag et al. 2016). Factors such as the socioeconomic status of the population of a given area, demographics and environmental awareness affect the quantity of MSW generated during a given year (Noori et al. 2009) and are caused by the rapid technological development and population growth experienced since the Industrial Revolution, resulting in an increase in the amount of generated waste (Adamcová and Vaverková 2016).

Effective waste strategies depend on local solutions and social awareness. The accumulation of waste in the environment raises social awareness due to the issues it causes (Matsakas et al. 2017). Apart from negatively affecting the environment, the waste also constitutes economic, social and ecological issues and causes the destruction of the landscape. As a member state, Poland is obliged to maintain sustainable waste management by limiting the generated amount of waste and minimising the deposited MSW on the basis of Directive 2008/98/EC (Gharfalkar et al. 2015). In this respect, to increase recycling, the country is required to selectively gather at least a fraction of the following waste products: paper, plastic and glass (Pomberger et al. 2017).

Such actions aim at improving waste management, environmental protection and the sanitary and aesthetic qualities of an area. The analysis of the changes implemented at the community level is essential to the issue of waste management, both on the basis of legal regulations and their effects on the level of collected waste (Malinowski and Kopytko 2014). Although many communities have not developed new waste management practices and standards, some of them have managed to implement different solutions to waste problems in the mountain context (UNEP 2017).

The obligations of communities and waste carriers are not without significance, as they bear many costs related to the management of solid waste, including the transport and maintenance of rolling stock, maintenance of waste collectors and operation of waste recovery and disposal plants (Semernya et al. 2017).

Against this background, it is important to identify the trajectory and efficiency of the changes in waste management in communities located in regions of high value in terms of the protection of nature, such as in the Carpathian Mountains. These factors were investigated based on the results of the accumulated waste per capita in two different research periods. The per capita data are widely used to compare the intensity of MSW generation among different places (Abu Qdais et al. 1997; Zhang et al. 2010; Karak et al. 2012).

The aim of this study was to assess the factors affecting the variability in the accumulation of municipal waste. Three communities located in the south of Poland within the Carpathian Mountains are considered. The accumulation rates relate to the changes in waste management that occurred between 2012 and 2013 and between 2014 and 2015.

## Materials and methods

### Materials and methods

This paper discusses issues concerning the efficiency of the changes in municipal waste management in three large settlement units located in a mountainous region in Poland, which are distinguished by their unique environmental conditions and attractiveness to tourists. In the analysis, the data came from the communities of Krynica-Zdrój, Piwniczna-Zdrój and Stary Sącz for 2012 to 2015 and are based on (1) a questionnaire distributed to these municipalities, (2) the researcher’s own observations from field visits and (3) information from Statistics Poland ((SP) n.d.; e.g. the number of tourists and inhabitants). In addition, the indicators of waste accumulation per capita have been extended to include bulky, glass, plastic, metal, organic, paper, electrical and electronic equipment and other sorted wastes, which are not usually differentiated as indicators of waste accumulation and are rarely used. The data include the size of the population, number of tourists, number of households and cost of waste management in the community areas.

Descriptive statistics were used, including the minimum, maximum and average. Moreover, for analytical purposes, except for the mentioned data, the amounts of selectively (segregated waste) and non-selectively collected waste (mixed waste) in individual communities were also used to determine the accumulation of waste per capita, considering the period before and after the changes related to the introduction of Directive 2008/98/EC into Polish legislation. It was assumed that the data from 2012 to 2013 fell into the period before the changes, while the years from 2014 to 2015 fell into the period after the changes.

### Characteristics of the communities

The urban-rural communities of Krynica-Zdrój (49° 25′ 58″ N, 20° 58′ 02″ E), Piwniczna-Zdrój (49° 43′ 33″ N, 20° 71′ 67″ E) and Stary Sącz (49° 33′ 49″ N, 20° 38′ 05″ E) are located in Nowosądecki County, which is situated in the south-eastern part of Małopolskie Voivodeship in the south of Poland. The communities are located in the mountains of the Sądecki region, which are separated by the Dunajec river and its tributaries (the Poprad river with sources in Slovakia and Kamienica). The region is dominated by the following mountains: the Beskid Sądecki, the Low Beskids and the Island Beskids.

The spa community of Krynica-Zdrój is situated on the northern side of the Carpathian Mountains in the east part of the Beskid Sądecki and on the western edge of the Low Beskids. It is a spa, resort and tourist destination with numerous sanatoriums and heath resorts due to its medicinal water sources. It is situated in the Beskid Sądecki within the area of the Poprad Landscape Park near the border of Slovakia. Most buildings in the community are approx. 550 to 1000 m above sea level (asl) in height, and some sanatoriums and private buildings are located well above 650 m asl.

The community of Piwniczna-Zdrój focuses on tourism and recreation. It has many holiday, health and recreation resorts and sanatoriums. The community area extends from an altitude of 400 to 1140 m asl. The community of Stary Sącz is one of the oldest settlement units of Poland, which also attracts tourists. It is located in Sądecka Valley in a fork between two mountain rivers (Poprad and Dunajec). These last two municipalities were founded in the twelfth century.

### Analysis of system solutions for waste management

Before the changes that resulted from Directive 2008/98/EC, in the community of Krynica-Zdrój, MSW was selectively collected to a small extent. In this period, before changes did not collect Waste Electrical and Electronic Equipment (WEEE) and organic waste from household. Other selectively collected wastes like ash, hazardous waste, concrete and demolition waste seldom were collected. After these changes, in accordance with the waste hierarchy (Gharfalkar et al. 2015), the obligation of sorting was introduced according to a minimum division of paper and paperboard, glass, metals and biodegradable waste. However, it was still possible to collect waste on a non-selective basis. After the changes, the collection of organic waste in bags was organised, despite the fact that it was possible for the property owner to compost such waste. The frequency of waste collection within the community, depending on the type of building (most often multi-family houses), was as follows:

- Sorted waste was collected one to four times a month.
- Mixed waste was collected two to four times a month.
- The frequency of waste collection within the community was as follows:
- Sorted waste was collected one to four times a month, depending on the type of building (most often multi-family houses);
- Mixed waste was collected two to four times a month, depending on the type of building (most often multi-family houses).

In Poland, before changes in waste management resulting from Directive 2008/98/EC, the most waste was collected as mixed waste, which was taken to landfill sites or sorting plants.

Within the community, the collection of bulky waste was organised at least twice a year. In 2014 to 2015, Piwniczna-Zdrój imposed an obligation to pay a “waste” fee on all inhabitants. Both sorted and unsorted MSW were collected in bags by the inhabitants. Such waste was collected at least once a month and twice a month in the summer.

An obligation to pay the waste fee was also imposed on all inhabitants in Stary Sącz after the introduction of the changes in waste management. The waste was collected according to the following frequencies:

- Sorted municipal waste was collected once a month.
- Mixed municipal waste was collected once or twice a month, depending on the season.

Additionally, within the community, ashes from furnaces were collected once a month during the 7 months requiring heating. The collected waste was exported outside the communities for further disposal because there was no installation for waste recovery and deposition in this mountainous area.

## Results

### The size of the areas of the communities

The Piwniczna-Zdrój community covers the largest area at 383 km^2^. The smallest area is the Krynica-Zdrój community at 145.13 km^2^. The area of the largest community is more than twice as large as the areas of the Stary Sącz (165.6 km^2^) and Krynica-Zdrój communities.

### Inhabitants and tourism

Table 1 presents a summary of the number of tourists and inhabitants in the three communities during 2012 to 2013 and 2014 to 2015. During the whole period, in Krynica-Zdrój, the greatest number of tourists (23,415) occurred in 2015 and the lowest (13,763) occurred in 2014. The highest average was 18,352.5. During 2012 and 2013, the number of tourists decreased by 4509, and during 2014 and 2015, there was an increase of 9652 tourists. In general, the highest increase in tourists was 3045, which was noticed from 2012 to 2015.

**Table 1:** The number of inhabitants and tourists in the three communes located in mountain region of southern Poland, with division into the period before and after the changes in waste management in the years 2012–2013 and 2014–2015, including descriptive statistics (minimum, maximum, average) (SP, 2012–2015)

Commune	Years				Statistic value		
	2012	2013	2014	2015	Min	Max	Average
Tourists (person)							
Krynica-Zdrój	20,370	15,861	13,763	23,415	13,763	23,415	18,352.25
Piwniczna-Zdrój	1039	1362	2162	2561	1039	2561	1781.00
Stary Sącz	36	238	418	939	36	939	407.75
Inhabitants (person)							
Krynica-Zdrój	16,980	16,991	16,862	16,858	16,858	16,991	16,923
Piwniczna-Zdrój	10,688	10,683	10,667	10,673	10,667	10,688	10,678
Stary Sącz	23,319	23,390	23,396	23,445	23,319	23,445	23,388

In Piwniczna-Zdrój, the lowest number of tourists was 1039, which occurred in 2012, and the highest was 2561, which occurred in 2015. Before the changes in waste management, there was an increase of 323 tourists. Similarly, after the changes, an increase of 399 tourists occurred. From 2012 to 2015, the increase in the number of tourists was 1522.

In 2012, the number of people visiting Stary Sącz was fairly low at 36, and the highest was recorded in 2015 at 939. The number of tourists increased by 202 and by 521 in the first and second research periods, respectively. During the whole research period, within the Stary Sącz community, there was an increase of 903 tourists, which were both the lowest increase and the average.

In Krynica-Zdrój, during the whole research cycle, the highest population of 16,991 occurred in 2013, which is before the changes to waste management and the lowest population (16,858) occurred in 2015 after the changes. In the first analysed period, the population increased by 11 people, whereas a decrease of four people was noticed in the second period. Generally, in this community, there was a decrease of inhabitants by 122 over the entire period.

In Piwniczna-Zdrój, the highest population of 10,688 was in 2012, and the lowest population at 10,667 occurred in 2014. In the first year (out of the 2 years), the number of inhabitants only decreased by five people. In the second year, the population increased by six people. In aggregate, a decrease by 15 people occurred within the area of the community. The lowest average of 10,677.8 also occurred there.

In the research period, in Stary Sącz, the highest population of 23,445 occurred in 2015, with the highest average of 23,388. There was an increase of 71 people in the first research period and an increase of 49 people in the second research period. This community is the only one that experienced an increase in its population, which was increased by 126 people.

### Households

**Fig. 1:**
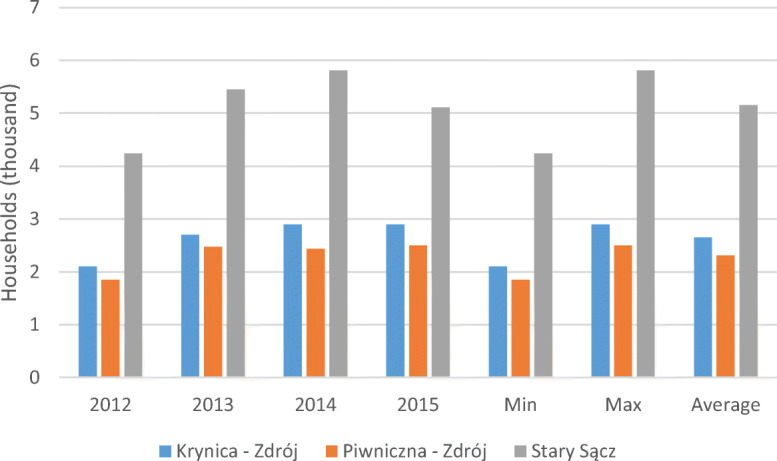
The number of households in the three communes located in mountain region of southern Poland, with division into the period before and after the changes in waste management in the years 2012–2013 and 2014–2015, including descriptive statistics (minimum, maximum, average)

The data presented in Fig. 1 include the number of households in the three communities from 2012 to 2015. The number of households in the community of Krynica-Zdrój in the first research period increased by 603. In the second period, the values were the same at 2901, which was also the lowest during the whole research period. The increase in the number of households in the community was 801.

In Piwniczna-Zdrój, the number of households in 2012 was 1851, which was the lowest in the research period. The highest number of households (2498) occurred in 2015, and the lowest average was 2313.50. Before and after the changes, the increases in the number of households were 621 and 65, respectively. The increase over the 4 years (2012 to 2015) was 647.

The highest increase in the number of households occurred in Stary Sącz (867). This community also had the highest average at 5152.75. In the mentioned community, it was noted that only an increase in the number of households occurred.

### Amount of municipal solid waste

Table 2 presents a summary of the collected MSW. The selectively collected municipal waste and the waste accumulation per capita within the areas of the three communities in the years 2012 to 2015 are considered.

**Table 2:** The amount of municipal solid waste taking into account selectively and non-selectively collected waste accumulation and rate per capita in the three communes located in mountain region of southern Poland, with division into the period before and after the changes in waste management in the years 2012–2013 and 2014–2015, including descriptive statistics (minimum, maximum, average)

Commune	Years				Statistic value		
	2012	2013	2014	2015	Min	Max	Average
Amount of collected MSW (Mg)							
Krynica-Zdrój	5872.25	6600.02	7569.17	6583.00	5872.25	7569.17	6656.11
Piwniczna-Zdrój	1174.40	1333.90	1353.06	1604.10	1174.40	1604.10	1366.37
Stary Sącz	1327.30	1810.43	5382.79	5496.57	1327.30	5496.57	3504.27
Amount of selectively collected MSW (Mg)							
Krynica-Zdrój	1782.09	1712.42	1871.37	1912.10	1712.42	1912.10	1819.50
Piwniczna-Zdrój	491.40	370.30	165.20	211.50	165.20	491.40	309.60
Stary Sącz	96.50	751.54	2709.49	2797.27	96.50	2797.27	1588.70
Amount of collected MSW (kg per capita^.^per year)							
Krynica-Zdrój	345.83	404.19	452.74	390.68	345.83	452.74	398.36
Piwniczna-Zdrój	112.82	123.88	126.83	148.04	112.82	148.04	127.89
Stary Sącz	68.24	73.79	210.30	220.62	68.24	220.62	143.24
Selectively collected waste (kg per capita per year)							
Krynica-Zdrój	104.95	100.78	110.98	113.42	100.78	113.42	107.54
Piwniczna-Zdrój	45.98	34.66	15.49	19.82	15.49	45.98	28.99
Stary Sącz	4.14	32.13	115.81	119.31	4.14	119.31	67.85
Non-selectively collected waste (kg per capita per year)							
Krynica-Zdrój	240.88	287.66	337.91	277.07	240.88	337.91	285.88
Piwniczna-Zdrój	63.90	90.20	111.36	130.48	63.90	130.48	98.98
Stary Sącz	52.78	45.27	114.26	115.13	45.27	115.13	81.86

In the first 2 years of the research period, the total amount of collected waste in the community of Krynica-Zdrój increased by 727.77 Mg, whereas in the next 2 years, there was a decrease of 986.17 Mg. In general, there was an increase in the weight of the waste by 710.75 Mg. The average was 6656.11 Mg, which was the highest. In Krynica-Zdrój, the weight of the selectively collected waste decreased by 69.67 Mg from 2012 to 2013, but in the second period, an increase of 40.73 Mg was noted, with the highest average of 1819.50 Mg. The largest amount of collected waste was noted in 2014 at 7569.17 Mg in Krynica-Zdrój. The amount of selectively collected waste in this commune also increased.

In Piwniczna-Zdrój, during the entire period, the amount of collected MSW increased by 429.70 Mg. The Stary Sącz community is also characterised with an increase in the amount of collected waste by 483.13 Mg in the first analysed period, with this value being lower by 113.78 Mg in the second period. This community is distinguished by the highest increase in waste, at 4169.27 Mg, between 2012 and 2015. The lowest amount of waste at 1174.40 Mg was observed in 2012 in Piwniczna-Zdrój. Piwniczna-Zdrój experienced a decrease by 121.10 Mg of the collected waste in the first period, and an increase by 46.30 Mg in the second period. This community is distinguished by exhibiting the largest decrease in the amount of waste by nearly 280 Mg from 2012 to 2015 and experienced the lowest average of 309.60 Mg.

In Stary Sącz, the highest increase of selectively collected waste (by 665.04 Mg) occurred in the years 2012 to 2013. There was a lower increase (87.78 Mg) from 2014 to 2015. The total amount of selectively collected waste from 2012 to 2015 confirmed the highest increase (2700.77 Mg). The quantitative data of the collected waste confirms that the community of Stary Sącz achieved the two most extreme values at 96.50 Mg and 2797.27 Mg.

### Indicators of waste accumulation

#### Mass accumulation rates

The municipal waste mass accumulation rate per capita per year in Krynica-Zdrój from 2012 to 2015 increased by 44.84 kg, with the highest average at 398.36 kg. The highest value was in 2014 at 452.74 kg for the whole research period. Moreover, from 2012 to 2013, the rate increased by 58.36 kg and decreased by 62.06 kg from 2014 to 2015.

In Piwniczna-Zdrój, the lowest value was 112.82 kg in 2012, and the highest value was 148.04 kg in 2015. The value increased by 11.06 kg and by 21.21 kg before and after the changes in waste management, respectively. Within 4 years, in Piwniczna-Zdrój, the increase in the rate per capita of waste accumulation was 35.22 kg, which was the lowest increase. The lowest average was 127.89 kg.

In Stary Sącz, the waste accumulation rate per capita showed the highest increase at 152.38 kg. Before the changes in waste management, this rate increased by 5.55 kg. After the changes, it increased by 10.32 kg.

The other waste mass accumulation rate for Krynica-Zdrój from 2012 to 2015 ranged from 0.95 kg per capita per day to the highest rate of 1.24 kg per capita per day, confirming an increase of 0.12 kg, with the highest average at 1.09 kg. In the first analysis period, the rate increased by 0.16 kg, and in the second period, it decreased by 0.17 kg, when considering the annual data.

In Piwniczna-Zdrój, the lowest rate per capita was 0.31 kg in 2012, and the highest was 0.41 kg in 2015. In the first 2-year period, the increase was 0.03 kg. In the second period, it was 0.06 kg. Within 4 years, the rate increased by 0.10 kg, which was the lowest increase observed on an annual basis.

In Stary Sącz, the rate per capita varied from the lowest value at 0.19 kg in 2012 to 0.60 kg in 2015, confirming the highest increase of 0.40 kg. Before the legislative changes in waste management, this value increased by 0.02 kg, whereas it increased by 0.03 kg after the changes.

The selectively collected waste accumulation rate per capita per year decreased by 4.17 kg and increased by 2.44 kg from 2014 to 2015, which was not a significant increase. The highest value of this rate, amounting to 113.42 kg for the entire period, occurred in 2015 in Krynica-Zdrój. In general, the rate increased by 8.47 kg, which was the highest average.

In Piwniczna-Zdrój, the situation is different. There was a decrease by 11.16 kg in the first research period, whereas an increase of 4.33 kg was noted in the second period. Between 2012 and 2015, there was a general decrease in the selectively collected waste accumulation rate per capita by 26.16 kg.

The highest increase (27.99 kg) in the first 2 years pertained to the community of Stary Sącz. In the second period, there was also an increase, but only by 3.50 kg in this area. The lowest rate amounted to 4.14 kg, which was noted in 2012. Despite this, the highest increase in the amount (115.17 kg) was noticed in the Stary Sącz community.

The highest non-selectively collected waste accumulation (337.91 kg) was noted in 2014 in Krynica-Zdrój. The highest increase per capita (from 2012 to 2013 at 46.78 kg) and the highest average (285.88 kg) both occurred in the same community. The greatest increase in this rate (66.58 kg) occurred in Piwniczna-Zdrój from 2012 to 2015. Before the legislative changes, in Stary Sącz, the lowest value of the rate was 45.27 kg. In addition, the lowest average was 81.86 kg.

In the examined communities, the value of waste accumulation per capita has generally decreased on selectively collected waste in the Piwniczna-Zdrój community.

Table 3 includes the municipal waste accumulation rate, selectively collected according to the eight types per capita on an annual basis with division into the two observation periods. The value of the accumulation rate per capita for glass waste, with the highest value of 37.11 kg, was observed in the Krynica-Zdrój community after the introduced changes. The highest average (34.26 kg) for glass waste was also noticed there. The lowest rate (1.22 kg) for glass waste occurred in Stary Sącz before the changes. The average glass waste for this community (15.08 kg) was also the lowest. In Krynica-Zdrój and Stary Sącz, rate increases were noticed for glass waste, although there was a decrease in the Stary Sącz community.

**Table 3:** The selectively collected waste accumulation rate per capita in the three communes located in mountain region of southern Poland divided into 8 types of waste with division into the period before and after the changes in waste management in the years 2012–2013 and 2014–2015, including descriptive statistics (minimum, maximum, average)

Commune	Years				Statistic value		
	2012	2013	2014	2015	Min	Max	Average
Glass waste (kg per capita^.^per year)							
Krynica-Zdrój	27.58	36.10	37.11	36.26	27.58	37.11	34.26
Piwniczna-Zdrój	23.75	12.07	5.62	8.35	5.62	23.75	12.44
Stary Sącz	1.22	7.24	25.12	26.74	1.22	26.74	15.08
Paper waste							
Krynica-Zdrój	59.63	28.01	17.11	15.74	15.74	59.63	30.12
Piwniczna-Zdrój	9.97	10.71	0.23	0.47	0.23	10.71	5.35
Stary Sącz	0.34	2.53	10.51	10.27	0.34	10.51	5.91
Metal waste							
Krynica-Zdrój	0.43	0.27	0.06	0.12	0.06	0.43	0.22
Piwniczna-Zdrój							
Stary Sącz		0.15	0.16		0.15	0.16	0.08
Plastic waste							
Krynica-Zdrój	17.31	24.28	32.72	36.30	17.31	36.30	27.65
Piwniczna-Zdrój	12.26	8.60	7.36	8.74	7.36	12.26	9.24
Stary Sącz	0.54	4.94	23.79	20.47	0.54	23.79	12.44
Bulky waste							
Krynica-Zdrój		8.81	16.57	14.53	8.81	16.57	9.98
Piwniczna-Zdrój		321.19	69.42	88.39	69.42	321.19	119.75
Stary Sącz	0.46	3.24	4.59	6.77	0.46	6.76	3.76
WEEE							
Krynica-Zdrój		0.09	0.34	0.16	0.09	0.34	0.20
Piwniczna-Zdrój		67.76	17.12	10.43	10.43	67.76	31.77
Stary Sącz		0.01		0.002	0.002	0.01	0.004
Organic waste							
Krynica-Zdrój		3.21	6.27	4.53	3.21	6.27	4.67
Piwniczna-Zdrój		0.60	0.34	0.22	0.22	0.60	0.38
Stary Sącz		5.10	22.64	17.36	5.10	22.64	15.03
Other selectively collected waste							
Krynica-Zdrój			0.79	5.78	0.79	5.78	2.19
Piwniczna-Zdrój			0.23			0.23	0.08
Stary Sącz	1.57	8.93	29.00	37.71	1.57	37.71	25.21

The highest value of the accumulation rate for paper waste during the whole period was in the community of Krynica-Zdrój in the amount of 59.63 kg in 2012. The lowest value for paper waste was 0.23 kg in 2014 in Piwniczna-Zdrój. The highest increase for paper waste was 9.93 kg in Stary Sącz. The lowest average (5.91 kg) and the highest decrease (43.89 kg) in paper waste occurred in Krynica-Zdrój, despite the highest average being 30.12 kg.

The analysis of the accumulation rate for metal waste within the 4 years showed both the highest value (0.43 kg) before the changes and the lowest value (0.06 kg) after the changes in Krynica-Zdrój. This community experienced both the highest average and highest decrease for metal waste. In Piwniczna-Zdrój, metal waste was not collected. In Stary Sącz, the researcher confirmed that there was collection 1 year prior to the changes and after the changes, with a low average.

The accumulation rate for plastic waste showed the highest value (36.30 kg) in 2015, the highest increase (6.97 kg) in 2012 to 2013 and the highest average (27.65 kg) in the community of Krynica-Zdrój. The lowest value and the largest decrease (3.32 kg) of plastic waste occurred in 2014 to 2015 in Stary Sącz, with the highest general increase of 19.93 kg.

Before the changes, the highest value of bulky waste accumulation was 321.19 kg in the Piwniczna-Zdrój community. This community is distinguished as having the largest area, whereas the highest increase (by 18.97 kg per capita) occurred in the period of 2 years after the changes, as bulky waste has been collected since 2013. Furthermore, in general, the highest increase (88.39 kg) and average (119.75 kg) occurred in this community. The lowest rate of 3.24 kg occurred in Stary Sącz before the changes, and the lowest average was 3.76 kg.

The accumulation rate for the WEEE confirmed the collection of such waste from 2013 to 2015 predominantly within the area of the first two communities (Krynica-Zdrój and Piwniczna-Zdrój). The highest amount (at 67.76 kg) occurred in Piwniczna-Zdrój. The lowest (0.002 kg) amount occurred in 2015 in Stary Sącz. The highest average of 31.77 kg occurred in the Piwniczna-Zdrój community, and the lowest (0.004 kg) occurred in Stary Sącz.

The results for organic waste accumulation within the area of the three communities in the mountainous region confirm an increase on average by 7.37 kg per capita between 2013 and 2015. The best result for organic waste accumulation was 22.64 kg, which was achieved in the third year in Stary Sącz, which had the highest increase of 17.36 kg and an average of 15.03 kg. The lowest result for the rate per capita (0.22 kg) was achieved in the fourth year in Piwniczna-Zdrój. It should be noted that a decrease in the accumulation rate was visible in each community in the second period.

The accumulation rate results for other selectively collected waste products, such as ash, hazardous waste, concrete and demolition waste, were analysed in Krynica-Zdrój and Stary Sącz. In the former, collection occurred only in the 2 years following the introduced changes, while in the latter, collection occurred within 4 years. The lowest result of 0.23 kg was achieved in 2014 in Piwniczna-Zdrój, and the highest result of 37.71 kg was achieved in Stary Sącz in 2015, with the highest increase at 28.78 kg and an average of 25.21 kg. An increase in the amount of separately collected waste occurred in the community of Stary Sącz.

#### Waste accumulation by area

**Fig. 2:**
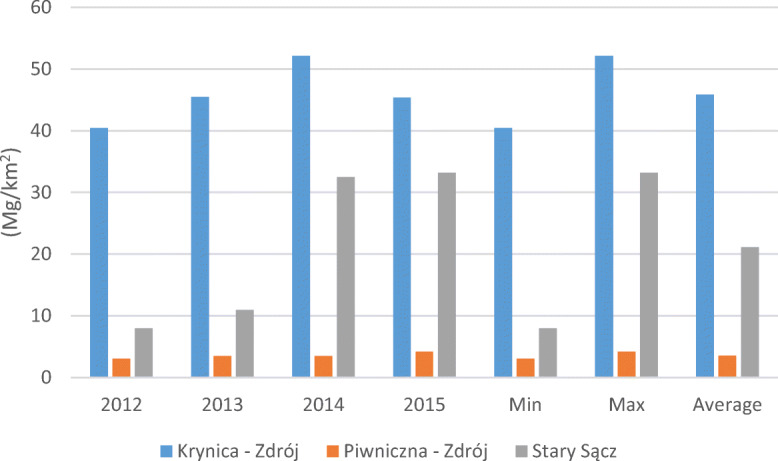
The waste accumulation rate per unit area in the three communes located in mountain region of southern Poland, with division into the period before and after the changes in waste management in the years 2012–2013 and 2014–2015, including descriptive statistics (minimum, maximum, average)

The results for waste accumulation per unit of area in the communities show an increase from 2012 to 2015 (Fig. 2). The highest value of the rate (52.15 Mg/km^2^) was observed after the changes, and the highest increase (5.02 Mg/km^2^) was observed in the period before the changes. The highest average at 45.86 Mg/km^2^ was in Krynica-Zdrój. The lowest value of the rate (amounting to 3.07 Mg/km^2^) pertained to the period before the changes in Piwniczna-Zdrój in relation to the lowest average of 3.57 Mg/km^2^. It should be noted that the highest increase in the rate during the research period was recorded in Stary Sącz at 25.17 Mg/km^2^.

#### Waste accumulation per household

**Fig. 3:**
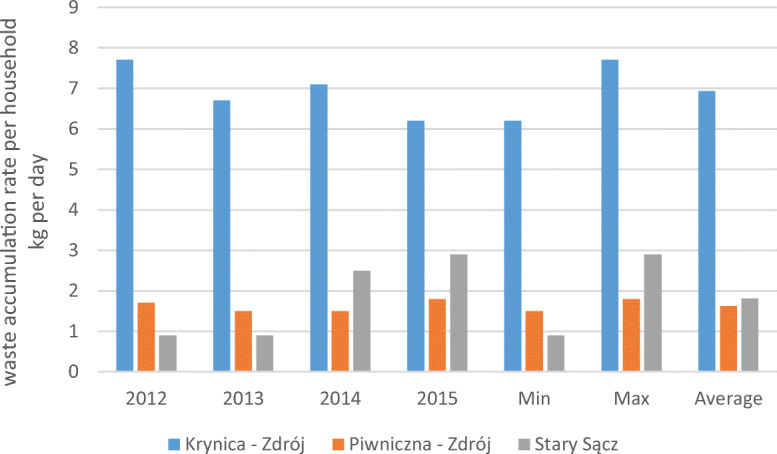
The waste accumulation rate per household in the three communes located in mountain region of southern Poland, with division into the period before and after the changes in waste management in the years 2012–2013 and 2014–2015, including descriptive statistics (minimum, maximum, average)

The results of daily waste accumulation per household in the analysed communities from 2012 to 2015 show an increase, with the exception of a decrease of 1.5 kg per household that occurred in the community with the greatest number of inhabitants (Fig. 3). The highest value of the rate in the amount of 7.7 kg per household occurred before the changes, and the highest average of 6.93 kg per household pertains to the community of Krynica-Zdrój. The lowest value of the rate, amounting to 0.9 kg per household, relates to the community of Stary Sącz, and it has stayed same for 2 years before the changes. In the community with the largest area, the daily lowest average was 1.62 kg per household.

Within 4 years, in the analyses, the municipalities demonstrated a decrease in the amount of waste accumulation per household.

#### Unit cost per capita

The cost of waste management in the analysed communities includes the costs of collection, transport, recovery and the disposal of waste (Table 4). The unit cost of waste management per capita, translated into euros in the communities on average increased by 15.41 EUR. The highest unit cost in the amount of 40.56 EUR was noted in 2014 in Krynica-Zdrój. The increase in the cost at 22.16 EUR and the average at 26.49 EUR were also the highest in this community. In the first period, the highest increase was 8.15 EUR in Piwniczna-Zdrój, where the lowest value of 3.76 EUR and an average of 12.93 EUR were noted. Overall, the value of the unit cost per capita has risen in all examined communities.

**Table 4:** The unit cost of waste management per capita on the basis of the exchange rate EUR/PLN *=* 42,359 in the three communes located in mountain region of southern Poland with division into the period before and after the changes in waste management in the years 2012–2013 and 2014–2015, including descriptive statistics (minimum, maximum, average) (rss.nbp.pl/)

Commune	Years				Statistic value		
	2012	2013	2014	2015	Min	Max	Average
(PLN per capita per year)							
Krynica-Zdrój	59.18	64.72	171.81	153.04	59.18	171.81	113.29
Piwniczna-Zdrój	15.93	50.45	74.81	77.90	15.93	77.90	54.77
Stary Sącz	44.01	26.52	75.61	83.91	26.52	83.91	57.51
(EUR per capita per year)							
Krynica-Zdrój	13.97	15.28	40.56	36.13	13.97	40.56	26.49
Piwniczna-Zdrój	3.76	11.91	17.66	18.39	3.76	18.39	12.93
Stary Sącz	10.39	6.26	17.85	19.81	6.26	19.81	13.58

#### The highest values of examined indicators

Table 5 includes a matrix consisting of the 19 rates with the highest values. These were noted in the analysed communities before and after the changes in the legal regulations in waste management. Krynica-Zdrój was distinguished most because the maximum values were achieved most frequently (12 times) and included the values for the number of tourists; amount of municipal waste; accumulation per capita of municipal waste, glass, paper, plastic, metals, sorted and mixed waste; municipal waste per area; and household and unit costs per capita. Stary Sącz showed a smaller number (five) of rates with the highest values, including the size of the population, number of households, amount of sorted waste and accumulation rates pertaining to sorted waste, other sorted waste and organic waste. In Piwniczna-Zdrój, the number of highest rates was the lowest (two) and covered the accumulation rates of bulky waste and WEEE. Before the changes in waste management, the highest values of the rates were observed five times in two communities (Krynica-Zdrój and Piwniczna-Zdrój). After the changes, there was a visible 13-fold increase in the number of the highest values. Out of these high values, the highest frequency (nine times) was found in the community most often visited by tourists, while the lowest frequency (five times) was observed in Stary Sącz, which has the highest number of inhabitants and households. The lowest frequency was achieved in 2012 (two times) and 2013 (three times), while the highest occurred in 2014 (seven times).

**Table 5:**
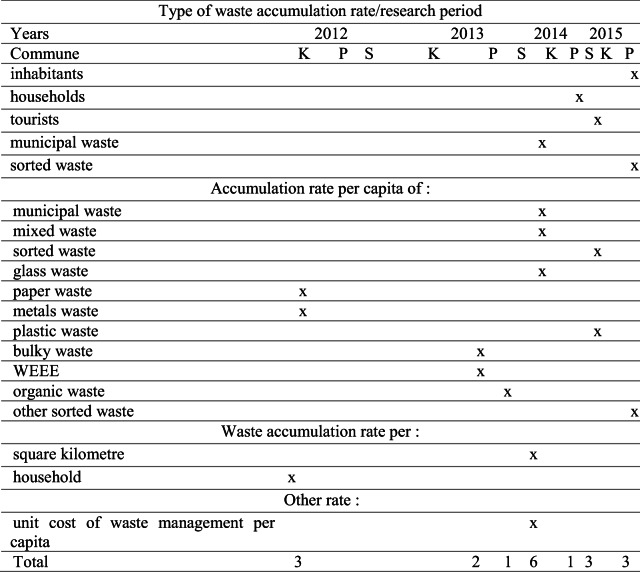
The highest value of inhabitants, households, tourists, municipal waste, sorted waste and accumulation rate per capita of municipal waste, mixed waste, sorted waste, glass waste, paper waste, metal waste, plastic waste, bulky waste, WEEE, organic waste, other sorted waste and waste accumulation rate per square kilometre, household and other rate of unit cost of waste management per capita achieved in the communes of the Krynica-Zdrój (K), Piwniczna-Zdrój (P) and Stary Sącz (S) located in mountain region of southern Poland with division into the period before and after the changes in waste management in the years 2012–2013 and 2014–2015

## Discussion

In areas of valuable natural beauty, particular attention is paid to the development of tourism and its effects on the environment. One of the factors increasing the amount of waste generated may be the development of tourism and, in particular, the number of tourists. Being attractive to tourists and well-known in Europe due to its natural beauty and tourist and health facilities, Krynica-Zdrój had the highest number of tourists at 23,415 and the highest increase at 9652 tourists between 2014 and 2015. Such a change could result from an increased interest in the tourist potential of the community. An increase is connected with the development of tourism, including winter tourism, which affects the economic stimulation of the community. The increase in the amount of waste related to tourism has also been noted by Kaseva and Moirana (2010), who detailed research on waste management in the region of Kilimanjaro.

Within a period of 4 years, the greatest amount of municipal waste (7569.17 Mg) was collected in Krynica-Zdrój after system changes to waste management. This community has the smallest area. The same relationship was demonstrated by Przydatek (2015). However, in the first period, the increase was 727.77 Mg, and in the second period, the decrease was 986.17 Mg. An increase in the amount of collected waste in this community is indicated by it having the greatest frequency of collection, which ranges from one to four times among the analysed communities. The number of inhabitants changed slightly after the changes in legislation. In the first research period, population growth occurred in Krynica-Zdrój and Stary Sącz. After the legislative changes, growth occurred only in the latter community. Such changes may have been caused by the decrease in the birth rate and the predominance of external migration. An important factor influencing the amount of waste generated was an increase in the number of tourists, despite the decrease in the number of inhabitants. Some research (Pons et al. 2009) has also shown that, in areas with strongly developed tourism, the generation of MSW may be variable and is heavily dependent on tourism, not just on the number of inhabitants. According to UNEP (2007), travel to mountainous regions may account for between 15 and 20% of global tourism.

Another factor that may affect the amount of waste accumulation is the number of households. The number of households in the analysed communities increased. In Stary Sącz, the number of households increased from 4240 to 5810, which was the highest increase (by 867 households) among the analysed communities. In the case of segregated waste, the highest increase (by 665.04 Mg) occurred from 2012 to 2013 in this community, where the amount of selectively collected waste from 2012 to 2015 showed the highest increase at 2700.77 Mg. This favourable result confirms the potential effect of an increase in the number of households on the selective accumulation waste in Stary Sącz. Such an increase of waste recovery as part of the selective collection of waste follows the trend in Japan (Hotta and Aoki-Suzuki 2014).

Multiple indicators of waste accumulation are helpful in recognising the direction of the changes taking place in waste management, considering the local geographic circumstances, socioeconomic factors and legal regulations. The highest value of waste accumulation per capita at 452.74 kg occurred in the first analysed year in the first community. A noticeable increase in the value of the considered indicators took place simultaneously with a decrease in the number of inhabitants and an increase in the number of tourists. This confirms the significant role of tourism in shaping waste accumulation per capita. Mixed-waste accumulation in the mountainous region was significantly higher than the value of 168 kg per capita per year presented by Łukasiewicz et al. (2017) in another community in Poland.

According to Mateu-Sbert et al. (2013), a strong relationship between waste and an increase in the population of tourists may exist. Such an increase occurred in this settlement unit (by 3045). An increase in the value of the rate accumulation to 380 kg per capita per year under the influence of tourism development was recorded in Turkey by Özbay (2015). A very close value of the accumulation rate of 481 kg per capita was achieved in the European Union (EU) (Eurostat 2014). For comparison, in some rural communities in central Poland, this indicator is much lower and fluctuates within the limits of 13–19.4 kg per capita per year (Przydatek 2015). In the periods before and after the legislative changes, an increase occurred in this waste accumulation rate on a daily basis in the communities, except for a decrease of 0.17 kg in Krynica-Zdrój after the legal regulations changed. In contrast, Taboada-González et al. (2010) showed a lower accumulation rate of 0.631 kg per capita per day in the rural communities in northern Mexico. This confirms that the changes in municipal waste accumulation per capita on both annual and daily basis retain the same volatility in their relationships.

In addition, compliance with the EU waste policy invokes measures that aim to reduce waste production per capita, while increasing the use of waste as a resource and making recycling attractive to both the private and public sectors (Vučijak et al. 2016**)**. In Stary Sącz, the analysed value varied from 0.19 to 0.60 kg and confirmed the highest increase (by 0.41 kg). For comparison, Przydatek et al. (2017) found a lower value, ranging from 0.23 to 0.26 kg per capita per day in the Małopolska Voivodeship within a similar period. This confirms the existence of the differentiation of waste accumulation between regions of the same country. Similar rate values of 0.30 to 0.70 kg on a daily basis per capita have been achieved in Asian countries (Kawai and Tasaki 2016).

However, Nenković-Riznić and Pucar (2014) conducted research into tourist settlements within Serbia and, on this basis, found an accumulation of waste per capita ranging from 26.4 to 163.8 kg, which significantly differed from the accumulation demonstrated within the area of the analysed community. Other indicators have also exhibited a variation in the waste accumulation rate. The highest value of the segregated waste accumulation at 113.42 kg for the entire period occurred in 2015. The highest value of the mixed-waste accumulation rate per capita at 337.91 kg was noted in 2014. This occurred in the same community, which experienced an increase in the number of tourists and a decrease in the number of inhabitants. These values are higher than the value indicated by Dahlén et al. (2007) in Sweden at 100 kg per capita per year. The comparable values of the accumulation indicators clearly underline the significant role of tourism in shaping the value of the waste accumulation rate in areas with unique environmental conditions.

Between 2012 and 2015, the paper waste accumulation rate increased to 9.93 kg, with an increase of the number of inhabitants by 126 people in Stary Sącz analysed community, which is in line with the increase in selectively collected waste and is particularly noticeable in the second research period. Similarly, Matsumoto (2011) demonstrated a correlation between an increase in this rate and the number of inhabitants. The analysis of the accumulation rate of metal waste per capita within the period of 4 years showed the highest value of 0.43 kg before the legislative changes in the Krynica-Zdrój community with the highest number of tourists. In other communities, the values were irregular due to the lack of the systematic accumulation of such waste resulting from price attractiveness and the possibility of individual sale for metal waste.

Buenrostro and Bocco (2001) considered the share of metals in municipal waste to be significant. This is why Ioana and Semenescu (2013) focused on this part in their research, finding a lack of respect for the principle of sustainable development in raw-material waste management. The value of the indicator relating to the waste accumulation rate per capita was the highest, in the amount of 36.30 kg and had the highest increase (by 6.97 kg) in the first period in Krynica-Zdrój. The values of these rates are lower than those reported by Abel (2007), amounting to 71.4 kg per capita. In the USA, the value of this accumulation rate was approx. 2.2 kg per capita (Subramanian 2000). A noticeable increase in this ratio and an increase in the number of households and total amount of waste in the first 2 years in Krynica-Zdrój indicated a convergence but excluded the factor related to the change in the law. According to Özbay (2015), people generate significant amounts of this waste, and these quantities are increasing with the standards of living and number of tourists. It is a characteristic of the community with the largest area to achieve the highest average of bulky waste accumulation rate per capita (amounting to 119.75 kg). Evidence in the UK suggests that the greatest tonnage diversion can be achieved for bulky waste (Cox et al. 2010).

Another important indicator in terms of recognising the efficiency of waste collection in municipalities is the accumulation of WEEE. In this type of waste, the highest value of the accumulation was 67.76 kg in the Piwniczna-Zdrój community with the largest area. This value was higher than that reported by Alavi et al. (2015) at the level of 0.42 kg. The values of these indicators in the second period in the settlement unit with the largest area and with the lowest average number of households suggest a certain effect of changes favouring the development of selective collection.

The organic waste accumulation rate was recorded within the three communities for only 3 years. The best result of this rate per capita on an annual basis was 22.64 kg per capita per year, which was achieved in the third year in Stary Sącz. This may be because this community has the highest number of households within its area. It should be noted that this rate is lower than the rate reported by Adhikari et al. (2008), which ranged from 0.61 to 0.56 kg per day. Despite the visible decrease of this rate in the second period, a general increase is noted during the specified 3 years, with the highest increase of 17.36 kg in the Stary Sącz community.

According to Metin et al. (2003), this organic waste represents a significant share in the overall mass of MSW. These values may indicate the possibility of waste management by inhabitants in their own way because the municipality has an urban-rural character. The changes that indicate the gradual involvement of the inhabitants in the collection of organic waste should be considered positive in this context. The progressive collection of waste management in the two communities, namely Krynica-Zdrój and Stary Sącz, is indicated by the accumulation of other waste collected per capita, including ash, hazardous waste, concrete and demolition waste. The highest value of the accumulation rate of other waste collected selectively in annual terms per capita occurred after the legislative changes and amounted to 37.71 kg in Stary Sącz, which had the greatest number of households. This result corresponds to a daily value of 0.10 kg and deviates from the demonstrated value in the range of 0.6–2.5 kg per capita, according to Buenrostro and Bocco (2001). In the community with the highest number of tourists, the rate of other waste accumulation was noticed only after the introduction of the legislative changes, demonstrating that such waste had not been collected earlier. Within the 4-year period in Stary Sącz, the highest increase was 28.78 kg per capita, resulting from an increase during the two research periods. As in the case of the other indicators, this indicates a positive effect of the amended legal requirements on limiting the amount of waste deposited.

It was important to evaluate the rate of waste accumulation per area. The highest increase in the waste accumulation rate per unit of area (5.02 Mg/km^2^) occurred before the waste management changes. The highest value of the waste accumulation rate per unit of area was 52.15 Mg/km^2^ after the changes in the community of Krynica-Zdrój. This value was higher than the value indicated by Przydatek (2016), which was within the limits of 6.2–13.4 Mg/km^2^ and was achieved within the area of the communities in the south of Poland. Such a result may be related to the numbers of households, inhabitants and tourists, which showed fluctuations despite the unremarkable area covered by this community. From the perspective of the municipality, it seems important to record the waste accumulation rate per household per day. In the waste accumulation rate per household, the highest rate was achieved in the first community, in the amount of 7.7 kg per household per day in 2012. This accumulation rate was greater than that demonstrated by Buenrostro and Bocco (2001) and by Sujauddin et al. (2008), with values from 1.3 to 2.71 kg per household per day. Such variability of the rate values suggests that the generation of solid waste in households is inflexible in relation to the population (Dahlén et al. 2007; Adhikari et al. 2008).

One of the important elements of waste management is the cost. The unit cost of waste management per capita in individual communities increased on average by 15.41 EUR. The highest cost in EUR was noted in Krynica-Zdrój after the legislative changes. It should be emphasised that this community is characterised by the highest increase in the amount of waste (by 710.75 Mg) and the highest average (6656.11 Mg). This confirms that the cost of waste collection is a factor that is related to the amount of waste generated. The predominant value occurred in the community distinguished by the greatest interest from tourists. Lohri et al. (2014) related the increase in waste collection costs to an increase in waste disposal.

Generally, before the legislative changes to waste management, the highest values of the indicators were recorded in the two communities with the smallest and largest areas. After the changes, there was an increase in the number of highest values by more than 10 times. This confirms that one of the important factors for beneficial changes to the level of waste accumulation is the inclusion of EU law in the national law in the field of waste management. However, the size of the geographic area does not matter. Most often, the highest rates of waste accumulation occurred in the community most frequently visited by tourists. Frequently, they included indicators for the amount of municipal waste and the accumulation per capita of municipal waste, glass, paper and metal waste collected selectively and non-selectively in the area as well as the unit cost. Based on studies conducted by Sujauddin et al. (2008), Burnley et al. (2007) and Buenrostro and Bocco (2001), a link exists between the generation of solid waste and the composition of household waste and between important socioeconomic traits, such as demographics and the cost of waste management. The results suggest that local geographic and demographic variables, including socioeconomic factors, help explain the collection rates in the communities, as demonstrated by Hage and Söderholm (2008).

## Conclusions

Based on the factors in the analysis of municipal waste management within the areas of the three communities situated in the southern part of Poland in the region of the Carpathian Mountains, the following conclusions have been drawn:

- A significant factor of changes in the level of waste accumulation was the inclusion of the EU law in the national law in the field of waste management. After the mentioned changes, the amount of collected glass and other selectively collected waste increased.
- The changes in the waste regulations improved waste management effectiveness, which was confirmed by more than a 10-fold increase in the highest rate values after the changes, and systematised the waste collection according to six types of waste.
- After the system changes, within a period of 2 years, 12 rates showed high values: the numbers of inhabitants, households and tourists; amount of municipal and sorted waste; the accumulations of municipal waste, sorted waste, glass, plastic and other selectively collected waste; and the per unit of area and unit cost.
- The number of households was an important factor that increased the amount of separately collected waste (e.g. at over 2000 Mg in Stary Sącz).
- The selected indicators will be helpful in determining the causes and direction of changes in waste management in other mountain communities in the context of the noticeable increase of selectively collected waste accumulation per capita.
- An important factor influencing the amount of waste generated was an increase in the number of tourists, despite the decrease in the number of inhabitants in Krynica-Zdrój, which confirmed an increase of interest in the tourist potential. In this community, the highest amount of the municipal waste accumulation was 452.74 kg, and the selective waste accumulation rate was 113.42 kg per capita per year.
- The cost of waste collection was related to the amount of waste generated in only one community with the greatest interest from tourists. In this community, overall, the highest values of the indicators were recorded the most often (12 times, nine of which represented significant changes in waste management).
- Multiple indicators of waste accumulation (13 indicators) are helpful in recognising the direction of the changes taking place in waste management, considering the local geographic circumstances, tourism, legal regulations and socioeconomic factors that have confirmed the existence of the differentiation of waste accumulation between three communities of the same county.
